# Mental Health in Educational Communities in Chile After a Public Health Emergency: An Assessment of Schoolchildren and Their Caregivers

**DOI:** 10.3390/medicina62020279

**Published:** 2026-01-29

**Authors:** Mariela Andrades, Felipe E. García, Ryan Kilmer, Pablo Concha-Ponce, Cibelle Lucero

**Affiliations:** 1 Instituto de Investigación y Doctorados, Universidad Central de Chile, Santiago 8330507, Chile; 2 Departamento de Psiquiatría y Salud Mental, Facultad de Medicina, Universidad de Concepción, Concepción 4070386, Chile; felipegarciam@yahoo.es; 3 Department of Psychological Science, University of North Carolina at Charlotte, Charlotte, NC 28223, USA; rpkilmer@charlotte.edu; 4 Facultad de Psicología, Universidad de Talca, Talca 3460000, Chile; pablo.concha@utalca.cl; 5 Facultad de Medicina y Ciencias de la Salud, Universidad Central de Chile, Santiago 8330507, Chile; lucerocariceo@gmail.com

**Keywords:** education, schoolchildren, mental health, pandemic, caregivers, posttraumatic stress symptoms, posttraumatic growth

## Abstract

*Background and Objectives*: Public health emergencies, such as the COVID-19 pandemic, significantly impact individuals and families, particularly in educational settings. School closures and changes in daily routines reduced students’ opportunities for learning and social interaction, affecting their mental health. Caregivers also faced increased responsibilities and stressors. This study aimed to evaluate a predictive model of mental health outcomes—specifically posttraumatic stress symptoms (PTSSs) and posttraumatic growth (PTG)—in Chilean schoolchildren and their caregivers. *Materials and Methods*: A total of 489 students (48% female sex; aged 10–17) from educational communities in various Chilean cities participated in the study, along with their caregivers (aged 21–69; 86.5% female), including mothers, fathers, and guardians. Mental health variables were assessed through self-report instruments. Hierarchical linear regression and path analyses were used to evaluate predictive models for PTSSs and PTG in students. *Results*: The model predicting PTSSs in students was significant. Key predictors included female sex, aggressive behavior, coping strategies such as keeping problems to oneself, cognitive avoidance, and intrusive rumination, and caregiver PTSSs. The model for PTG was also significant, with predictors including active problem-solving, communication, a positive attitude, and deliberate rumination. These results indicate distinct psychological processes underlying negative and positive outcomes following trauma. *Conclusions*: The findings underscore the complexity of mental health outcomes among school-aged children and the influence of caregiver well-being. The study highlights the importance of supporting both students and caregivers through targeted interventions. Multi-level strategies addressing emotional regulation, communication, and coping mechanisms may foster resilience and psychological growth in educational communities facing the aftermath of public health emergencies.

## 1. Introduction

The coronavirus pandemic, which began in 2019 (COVID-19), was a rapidly spreading public health emergency that generated a significant emotional, social, and economic impact [1], including human losses [2]. In addition to its effects on physical health, the pandemic was a multidimensional stressor [3], bringing serious risk to mental health [4]. Quarantine and social distancing measures, public health strategies implemented to prevent the spread of the virus, meaningfully affected people’s daily lives, influencing their work, academic functioning, school experiences, leisure activities, and social relationships [1].

In Chile, recent research has highlighted that pandemic-related disruptions affected students’ perceptions of school violence and the management of school coexistence. Specifically, changes in school environments during and after the pandemic influenced social dynamics, increasing concerns about peer aggression and negatively impacting students’ sense of safety and psychological well-being. This underscores the multidimensional impact of the pandemic on children and adolescents, extending beyond physical health to affect their social contexts and development [5].

In Chile and Latin America, research on the effects of public health emergencies, such as the COVID-19 pandemic, on the mental health of students and their caregivers is limited. While there are studies that address the impact of the pandemic on the mental health of children and adolescents in Latin America [5], a deeper understanding is still needed of how these effects manifest in the Chilean context, particularly within educational communities. Therefore, this study aims to fill this gap and provide evidence to inform the development of support strategies.

These quarantine measures, forced isolation, loneliness, illness, fear of contagion and death of loved ones, contributed to the development or exacerbation of various psychological problems [6] effects attributed to the increase in stress and uncertainty for many [7]. Existing studies also suggest that some people developed psychological problems as a consequence of the pandemic [8]. Among the most common reactions were symptoms of stress, anxiety, depression [9], fear, loneliness, increased substance use [10] and post-traumatic stress [11]. Longitudinal studies have reinforced these findings, with reports of higher levels of anxiety and depressive symptoms relative to pre-pandemic functioning in the same population [12]. Research suggests that pandemic-related risk factors for post-traumatic stress disorder (PTSD) include being female [13], pre-existing emotional and behavioral problems [14], and being younger, with children and adolescents identified as one of the most affected groups [15]

Children and adolescents were highly exposed to biopsychosocial stressors related to the pandemic [16]. Lockdown and social distancing measures led to school closures and students staying at home. Studies suggest that isolation from peers and other people in their social networks severely affected youths’ mental health [17]. A meta-analysis found that approximately 48% of young people aged 0 to 18 years had experienced post-traumatic stress [18].

In Chile, recent research has shown that these pandemic-related disruptions affected students’ school experiences. Specifically, changes in school environments during and after the pandemic influenced social dynamics, increasing concerns about peer aggression and negatively impacting students’ sense of safety and psychological well-being. This underscores the multidimensional impact of the pandemic on children and adolescents, extending beyond physical health to affect their social contexts and development [5].

In considering changes to social and developmental contexts during the pandemic, children’s home environments are also salient. Indeed, during lockdown situations, parents or caregivers tended to interact most with children and adolescents [19]. In turn, parental or caregiver functioning, including the post-traumatic symptoms of parents or caregivers, could influence the symptoms of their children [20,21].

In the broader literature on the impact of adversity, evidence suggests that, in addition to potential adverse psychological effects in the short and long term, individuals may also experience positive changes as a result of the struggle undertaken to cope with the consequences of a highly stressful event or condition [22], a phenomenon referred to as post-traumatic growth (PTG; [23]). According to [22], for an event to generate PTG, a moderate level of distress must occur. Thus, one’s subjective perception of the severity of the event or condition, as well as the emotional and cognitive disruption and distress experienced, can contribute to the development of post-traumatic symptomatology and PTG [23,24,25,26]. In other words, the subjective severity of the event and its aftermath can lead to the development of post-traumatic symptomatology, PTG, or both [27].

However, not all people who experience potentially traumatic events will necessarily experience PTG; their likelihood of experiencing PTG is related, at least in part, to internal resources and factors such as the level of self-efficacy, sense of meaning, and optimism [28], as well as social and contextual factors, including their social support networks and family relationships [29]. Thus, in family contexts, parents and/or caregivers are considered important potential facilitators of PTG not only by providing material or instrumental support, but also by promoting a sense of belonging, understanding, and encouragement [30].

The adaptation of the PTG model in children and adolescents, as postulated by Kilmer (see, e.g., [25,31,32,33], underscores the potential contribution of child resources, as well as relational and other contextual variables, to PTG. Such factors include children and youths’ assumptions, beliefs, and perceptions about themselves, others, and the world, as well as qualities of the parent–child relationship and their own and others’ psychological adjustment. In support of these notions, studies have identified a relationship between the family environment and PTG in children, for example, following a natural disaster [34].

Overall, in addition to work focusing on the potential negative consequences of exposure to significant adversity, a substantial body of research shows that children and youth can cope and adapt effectively, e.g., [35], and that some children and adolescents may also experience positive changes and positive learning following significant life events [33], outcomes that were also observed during the COVID-19 pandemic [36]. For example, one study found that 45.6% of adolescents reported PTG during the COVID-19 pandemic [37].

In view of possible post-adversity trajectories, evidence suggests that a variety of factors contribute to PTSD or PTG following a stressful or potentially traumatic event [38]. For example, while the presence of repetitive intrusive thoughts is strongly related to PTSD, productive ruminative processes such as deliberate thinking are associated with well-being and PTG [39]. In the extant literature, evidence indicates that intrusive and deliberate rumination are central to the development of PTSD and PTG in children and adolescents [33,40,41].

Studies that have addressed coping strategies and their association with PTG and PTSD have reported that problem-focused strategies have been associated with favorable socioemotional adaptation in childhood, promoting PTG [41]. For example, results from a study of youth diagnosed with cancer found that those who used productive, acceptance-focused coping strategies reported higher levels of PTG than those who used avoidant coping strategies [42]. On the other hand, unproductive strategies (cognitive avoidance, behavioural avoidance, indifference, keeping the problem to oneself, and aggressive behaviour) are associated with lower psychological well-being and adjustment problems in childhood [43]. Caregivers also appear to play a role—research has shown that parents’ self-reported PTG can be a significant predictor of PTG in their children [44].

Some studies have evaluated PTG in parents of children with special health care needs. One of the first investigations on this topic assessed PTG in parents of children with chronic illnesses and estimated that approximately 62.7% of parents experienced at least a moderate degree of PTG [45]. This finding has been replicated in parents of children with autism spectrum disorders [46], cancer [47], and Down syndrome [48]. These studies’ researchers concluded that PTG was associated with better problem-solving ability, parenting skills, and mental resilience of parents [46,48].

In general, research that has assessed the associations of PTG and PTSD in caregivers and their children is sparse [49]. Typically, researchers have focused on children’s or parents’ experiences with PTSD or PTG separately [50] or have explored the influence of child-perceived parental factors on PTSD and PTG [51]. These approaches may preclude a deeper understanding of PTSD and PTG in children and the interdependent relationship between children and their parents.

Indeed, researchers have documented that the relationship between children and their parents may relate to children’s internalizing symptoms and social functioning in PTSD [52]. It is therefore plausible that PTSD and PTG covary in close relationships, such as between children and their parents (dyads), and that the PTSD-PTG relationship may be best understood from a family perspective [53]. Broadly, the available findings indicate that parental functioning may affect children’s positive adaptation following a disaster and highlight the need to assess the potential influences of parents and other sources of support in the child’s environment following trauma. Attending to such factors is essential for efforts to promote adaptation and facilitate PTG [44]. Investigating the influence of the primary caregiver on the mental health of their children exposed to a highly stressful event is relevant, as it could guide interventions that health professionals provide to children, their families, and their educational communities.

In view of this context, the present study is guided by two primary research questions: (1) What factors predict the development of post-traumatic symptoms (PTSSs) and PTG in children and adolescents during the COVID-19 pandemic? (2) To what extent do parents influence their children’s PTG and post-traumatic stress?

It is hypothesized that productive coping strategies and deliberate rumination significantly predict PTG, and that unproductive strategies and intrusive rumination significantly predict post-traumatic symptoms. In addition, it is hypothesized that factors related to specific characteristics of the pandemic context (infection of a family member, death of a family member/friend due to COVID-19, and having been infected), as well as sociodemographic (sex and age) and psychological variables, will moderate the relationship between the predictor variables and the dependent variables of PTG and PTSSs. Finally, we posit that parents’ posttraumatic symptomatology and PTG will significantly predict their children’s posttraumatic symptomatology and PTG.

## 2. Materials and Methods

### 2.1. Design

A descriptive and correlational design was used. The study was cross-sectional.

### 2.2. Participants

Participants included 489 children and adolescents, along with their primary caregivers, from five cities across northern, central, and southern Chile. In the child and youth subsample, 48.1% were girls, with ages ranging from 10 to 17 years (M = 12.90; SD = 2.04). Children with a severe neurological problem diagnosis were excluded. Among caregivers—adults directly responsible for the care of the participating children and adolescents—86.5% were women, and their ages ranged from 21 to 69 years (M = 41.47; SD = 7.56).

### 2.3. Student-Completed Measures

Post-traumatic symptomatology. The Children’s Posttraumatic Stress Disorder Symptom Scale (CPSS; [54]) assesses the presence and severity of PTSD symptoms in children and adolescents aged 8 to 18 years with a history of known trauma. The scale is based on the DSM-IV diagnostic criteria for PTSD. It consists of 17 items with Likert-type responses indicating the frequency of symptom manifestation for this disorder, ranging from 0 (never) to 4 (9 times or more). The total score ranges from 0 to 68, with higher scores reflecting higher levels of PTSD symptoms. The CPSS was validated in Chile [55], yielding a level of internal consistency similar to that of the original instrument (*α* = 0.91) and a 90.7% discrimination capacity of the scale regarding the presence/non-presence of PTSD, as established by clinical criteria. The cut-off score was set at 24 points, which indicates a probable diagnosis of PTSD [55].

Coping strategies. The 35-item Children’s Coping Scale [56] assesses coping strategies in response to daily childhood stressors across four areas: health, school, peers, and family. Items reflect problem-focused coping and unproductive coping, and respondents indicate the frequency with which they employ a given strategy (0 = never; 2 = often). Productive coping strategies include active solving, communicating the problem to others, seeking information and guidance, and maintaining a positive attitude. Unproductive strategies include indifference, aggressive behavior, keeping the problem to oneself, cognitive avoidance, and behavioral avoidance. In a study involving children exposed to a natural disaster [41], obtained internal consistencies were *α* = 0.87 for productive and *α* = 0.78 for unproductive strategies. Available findings suggest that the measured strategies are associated with indicators of health and psychological adjustment in childhood [56].

Posttraumatic growth. On the 10-item Posttraumatic Growth Inventory for Children—Revised (PTGI-C-R [32,57], respondents endorse the degree to which they have experienced different types of change (e.g., “I learned how nice and helpful some people can be,” “I know what is important to me better than I used to.”) on a 4-point scale from 0 (no change) to 3 (a lot). However, the scale was developed to reflect five domains of potential positive change, and the Chilean validation [57] established two factors: general change and spiritual change. This instrument has shown adequate internal reliability (*α* = 0.77) and temporal stability (*r* = 0.44) [32].

Intrusive and deliberate rumination. The five-item Rumination Scale for Children [40,58] assesses intrusive (e.g., “I think about it when I don’t mean to.”) and deliberate (“Sometimes I think about it to try to figure out why things like that happen.”) rumination using a four-point scale from 0 (“I don’t think about anything”) to 3 (“I think about this a lot”). In the study by Andrades and García [41], *α* = 0.94 was obtained for deliberate rumination and *r* = 0.71 for intrusive rumination.

Sociodemographic characteristics. Youth also reported their gender, age, grade, and household composition.

### 2.4. Caregiver-Completed Measures

Post-traumatic symptoms. On the 12-item expanded version of the Short Posttraumatic Stress Disorder Rating Interview SPRINT-E [59,60], parents or caregivers reported about their potential posttraumatic stress symptoms, using a scale ranging from 0 (nothing) to 3 (a lot). When using the translated version, Leiva-Bianchi and Gallardo [60], obtained a good internal consistency of *α* = 0.92. There was also evidence of concurrent and construct validity in the Chilean population.

Post-traumatic growth. The Posttraumatic Growth Inventory–Short Form (PTGI-SF; [61,62]) assesses multiple dimensions of PTG using 10 items, answered on a scale ranging from 0 (no change) to 5 (a very important change). García and Wlodarczyk reported a Cronbach’s alpha of 0.94 and evidence of concurrent and construct validity in the Chilean population.

Subjective severity of the event. Three questions developed by Alzugaray et al. [63] were adapted to the pandemic context and used to evaluate the perceived severity of the event (i.e., To what extent do you feel that the pandemic has altered your life?; To what extent do you rate the pandemic as a traumatic experience in your life?; To what extent do you think the pandemic has been serious?). Each question is answered on a scale ranging from 0 (not at all) to 4 (very much), with a maximum score of 12; higher scores indicate greater perceived severity. In the study by Alzugaray et al. [63], a Cronbach’s alpha of 0.84 was reported.

Sociodemographics and selected background. A questionnaire was developed for this effort to collect sociodemographic information (gender, age, nationality) and background related to the pandemic. The latter dichotomous (yes–no) items were if the respondent had been infected, if a family member had been infected, if a family member was hospitalized, or if a family member or friend died due to COVID-19.

### 2.5. Procedure

Data collection was carried out from August to December 2022. During that period, many of the restrictions implemented in 2020 and 2021 to contain the pandemic began to ease. The mass quarantines had ended in Chile, and students had returned to the classroom, but masking was mandatory, and they had to maintain interpersonal distance.

Telephone and email contact was established with educational centers to provide leadership with information about the project and to assess their interest in participating. Participants were selected from different regions, school types, and socioeconomic groups to obtain a sample as heterogeneous and representative as possible.

In each center, classrooms were randomly selected for data collection. Parents or caregivers from participating classrooms were informed of the study in advance, and their consent was obtained. Student consent was also requested. Participation was voluntary, and participants’ identities were kept strictly confidential.

The questionnaires were administered in person to children and adolescents in their respective classrooms, with support from psychologists, undergraduate and graduate students, and the classroom teacher, who had received prior training to ensure proper administration of the instruments.

Data were collected from parents or primary caregivers during meetings in various cities.

The project was reviewed and approved by the Committee on Ethics of Scientific Research de la Universidad Central de Chile, under Resolution No. 46/2020 (date: October, 20 2020).

### 2.6. Data Analysis

Study analyses were conducted using SPSS v.21 and AMOS SPSS 20.0. After descriptive analysis of the study variables, bivariate relationships were evaluated using Pearson’s r correlation coefficient. The models were then evaluated using hierarchical linear regression, after assessing the statistical assumptions.

After evaluating multivariate normality, a path analysis was performed using maximum likelihood estimation to estimate the model. *χ*^2^ was used as a goodness-of-fit index; however, because this index is sensitive to sample size, the quotient obtained from the division between *χ*^2^ and its respective degrees of freedom (*χ*^2^*/df*), the Comparative Fit Index (*CFI*), the Tucker–Lewis Index (*TLI*), the Root Mean Square Error of Approximation (*RMSEA*), with its respective confidence interval, and the Standardized Root Mean Square Residual (*SRMR*) were also used. A good fit is indicated when the following criteria are met: the *χ*^2^ yields a *p* > 0.05, the *χ*^2^*/df* does not exceed 5 points, the *CFI* and *TLI* exceed 0.95, the SRMR and *RMSEA* have a value less than 0.08, and the *RMSEA* confidence interval is less than 0.10 (Hair et al., 2010) [64].

To evaluate the mediations, a bias-corrected bootstrap estimation was performed (2000 bootstrap samples with a 95% confidence interval). In this case, mediation exists if zero is not included in the confidence interval [65], indicating that the indirect effect between two variables is significant.

Missing data were replaced with a Bayesian method.

## 3. Results

The data showed a multivariate normal distribution—the Mardia coefficient yielded a critical ratio of 2.88, which is lower than the limit proposed by Rodríguez and Ruiz [66] for the use of parametric estimation methods. Descriptive and internal consistency analyses were performed on the study variables. Table 1 shows that most variables had acceptable to good internal consistency, although values for the rumination subscales and some coping strategies subscales suggested low reliability. Because the scales have been used in previous research with Chilean children and youth and exceeded the minimum of 0.50 suggested as acceptable for research purposes [67], we retained the measures for study analyses.

Table 2 shows that correlations among study variables show that aggressive behavior (*r* = 0.42) and keeping the problem to oneself (*r* = 0.41), both unproductive coping strategies, were the strategies that correlated most highly with posttraumatic symptomatology in children; youths’ posttraumatic symptoms also correlated significantly with intrusive rumination (*r* = 0.51). Problem-focused strategies of positive attitude (*r* = 0.48) and active solution (*r* = 0.41) had the highest correlation with PTG. PTG correlated significantly with deliberate rumination (*r* = 0.31) in children. Caregivers’ posttraumatic stress symptoms correlated significantly with children’s post-traumatic stress symptoms (*r* = 0.16), aggressive behavior strategies (*r* = 0.13), cognitive avoidance (*r* = 0.13), and intrusive rumination (*r* = 0.11). Caregivers’ PTG correlated positively with children’s post-traumatic symptomatology (*r* = 0.14), cognitive avoidance strategies (*r* = 0.11), active solution (*r* = 0.11), and keeping the problem to oneself (*r* = 0.10).

The correlation between children’s PTSSs and PTG was inverse and significant (*r* = −0.28); the association between caregivers’ PTSSs and PTG was direct and significant (*r* = 0.35).

Among the sociodemographic variables, children’s age correlated negatively with children’s PTSSs (*r* = −0.12; *p* = 0.006), but not with caregivers’ PTSSs or children’s or caregivers’ PTG. When examining sex, the Student *t*-test was used to compare PTSSs (t = 5.685; *p* < 0.001; Cohen’s *d* = 0.53), indicating that girls (M = 25.98; SD = 14.12) reported more PTSSs than boys (M = 19.09; SD = 11.37). In the case of PTG in children (t = −3.463; *p* < 0.001; Cohen’s *d* = 0.32), girls (M = 16.78; SD = 5.80) endorsed lower levels than boys (M = 18.69; SD = 6.26).

When exploring the role of COVID-19-related variables, the analyses showed no differences in PTSSs or PTG between those who were infected and those who were not infected, nor between those who suffered the infection of someone close to them (or not), nor between those who suffered the death of someone close to them and those who did not experience a COVID-19-related loss, neither in children nor in their caregivers.

The evaluation of the statistical assumptions for performing multiple linear regression indicated that the data met the criteria of linearity, independence of residuals, homoscedasticity, normality of residuals, and absence of multicollinearity.

A hierarchical multiple linear regression model was performed to evaluate predictors of PTSSs and PTG in children. The first step included sex and age. The second step included psychological variables, i.e., coping strategies and rumination. In the third step, subjective severity, PTSSs, and PTG of caregivers were included.

The final model for PTSSs was significant, *F*(15, 473) = 28.886, *p* < 0.001, with an Adjusted *R*^2^ = 0.46, implying that the model predicts 46% of the variance in the dependent variable. Predictors of PTSSs are having aggressive behavior (*β* = 0.23), keeping the problem to oneself (*β* = 0.20), using cognitive avoidance (*β* = 0.13), experiencing intrusive rumination (*β* = 0.39), and parental posttraumatic symptoms (*β* = 0.08); the coping strategy of trying to be positive (*β* = −0.18) predicts fewer PTSSs.

The final model for PTG was also significant, *F*(15, 473) = 20.126, *p* < 0.001, with an Adjusted *R*^2^ of 0.37. Predictors of PTG are attempting to identify an active solution (*β* = 0.16), communicating with others (*β* = 0.11), positive attitude (*β* = 0.16), and deliberate rumination (*β* = 0.20); responding with aggressive behavior (*β* = −0.09) was associated with lower PTG. Table 3 summarizes the PTSS model. Table 4 summarizes the PTG model.

**Figure 1:**
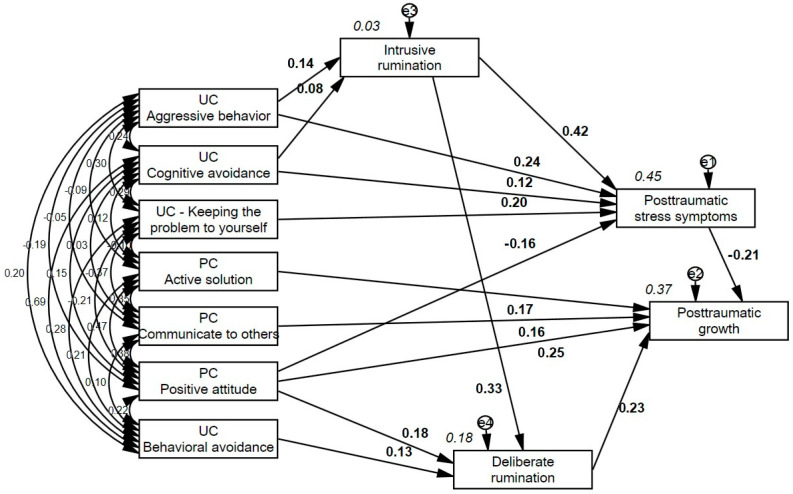
Final model of symptomatology and posttraumatic growth of children and adolescents during the COVID-19 pandemic (PC = problem-focused coping; UC = unproductive coping; PTSS: Posttraumatic stress symptomatology; PTG: Posttraumatic growth), (weights are standardized).

As a final step, we evaluated the hypothesized model. Results suggested adequate goodness of fit: *χ*^2^ = 171.735, *df* = 39, *p* < 0.001; *χ*^2^*/df* = 4.40; *CFI* = 0.98; *TLI* = 0.96; *RMSEA* = 0.041, *CI* = 0.040 to 0.042; *SRMR* = 0.028. The model was then respecified by removing nonsignificant paths or those with loadings below 0.08; these were Deliberate Rumination → PTSSs Children (*β* = 0.04), PTSSs Caregivers → PTSSs Children (*β* = 0.05), and PTG Caregivers → PTG Children (*β* = 0.04). Modification indices did not indicate any other significant paths to add to the model. These modifications resulted in a better-fitting and more parsimonious model (*χ*^2^ = 71.207, *df* = 19, *p* < 0.001; *χ*^2^*/df* = 3.75; *CFI* = 0.99; *TLI* = 0.97; *RMSEA* = 0.023; *CI* = 0.023 to 0.023; *SRMR* = 0.016). This final fitted model is shown in Figure 1

Finally, mediation analyses showed that intrusive and deliberate rumination mediated the relationship between coping strategies and children’s PTSSs and PTG (see Table 5).

## 4. Discussion

This study evaluated a predictive model of PTSSs and PTG during the COVID-19 pandemic. It assessed the associations between sociodemographic, pandemic-related, and psychological variables and PTSD and PTG in children and adolescents and their primary caregiver. The results of this study contribute to a deeper understanding of the processes that lead to PTG and PTSD in children and their caregivers after a potentially traumatic experience such as the COVID-19 pandemic. They also provide evidence of the importance of coping strategies and different rumination processes as predictors of these post-trauma responses.

Analysis of the effect of demographic variables in this sample suggested that, for children, younger age and female gender were associated with higher PTSSs. This may suggest that younger children have difficulty understanding what is happening in potentially traumatic situations or have a more limited capacity to cope and respond in such circumstances (e.g., [68]). It is also likely that in the case of younger children, changes in routines and confinement have a greater impact on their daily lives and activate psychological mechanisms that lead to higher levels of distress, such as intrusive rumination and post-traumatic stress symptoms [69]. The finding that girls reported more PTSSs than boys is consistent with previous studies in children and adolescents [70], in which a greater tendency to experience and express distress, anxiety, and depression associated with stress or adversity is observed. Some have attributed these observed differences to socialization processes and variability in how caregivers/parents may respond to children in the context of adversity [71].

That boys endorsed higher levels of PTG than girls is consistent with a recent study that also found higher PTG in adolescent boys than in girls during the COVID-19 pandemic [19]. However, among young adults, women reported higher PTG during the pandemic than men [72]. Some evidence suggests that girls may recover more slowly from stressful events than boys due to the negative cognitive appraisals of threats that they continue to make, and this ongoing distress could be related to PTG [73]. Further studies are needed to investigate these sex differences.

Unexpectedly, no differences were found based on the assessed COVID-19-related variables. It appears that, at least in this sample, those who participated in the study experienced sufficient disruption and cumulative or ongoing distress as a result of the pandemic that the specific circumstances assessed did not contribute to differential outcomes.

Caregivers’ functioning, specifically their PTSSs, is related to their children’s PTSSs. This is consistent with other research supporting the potential negative impact of parental PTSSs, contributing to their children’s PTSSs [74]. While research on potential levels of PTSSs or PTSD among caregivers and children in the context of a pandemic is scarce, evidence from cross-sectional surveys indicates that greater anxiety, depression, and post-traumatic stress among caregivers during lockdown was associated with greater emotional difficulties in their children [75]. It is possible that symptoms related to emotional dysregulation can overwhelm caregivers and interfere with their ability to respond sensitively to their children [76]. Recent findings indicate that withdrawal after a traumatic experience may be an equally common parental response to such situations [77]. In the broader literature, findings suggest that a cold or tense family environment can contribute to low self-compassion [78]. Adolescents with low self-compassion may experience more emotional distress, problematic rumination, and trauma-related negative emotions [79], which may lead to more PTSD symptoms [80]. Parental family and social support were also incorporated as explanatory variables. Therefore, those who lack these protective factors are more likely to present with PTSD-associated symptoms.

Caregivers’ PTG was not associated with that of their children. However, previous studies that evaluated the relationship between family environment and PTG in children after a natural disaster [34] found that caregivers’ self-reported PTG was a significant predictor of PTG in their children, suggesting that social processes play a role in the development of PTG in childhood and youth and that the family context could facilitate positive cognitive processing and activate a sense-making process [38]. Longitudinal studies are necessary to investigate and enhance the understanding of this relationship.

Correlation analysis showed that unproductive coping strategies were associated with PTSSs. Strategies such as engaging in aggressive behavior, keeping the problem to oneself, and cognitive avoidance appear to be related to greater stress development after an event such as the pandemic, perhaps because denying the severity of a problem and trying not to think about it can lead to more recurrent and intrusive memories of the trauma [81]. The students’ PTSSs also correlated with intrusive rumination. Evidence shows that, after a traumatic event, involuntary and unwanted thoughts about the experiences can increase the likelihood of developing PTSSs [61].

Consistent with expectations, problem-focused strategies such as maintaining a positive attitude and actively seeking solutions related to PTG are effective. Productive strategies imply that children and adolescents maintain a sense of control, leading them to confront the consequences of a stressful event rather than avoid them, thereby facilitating a sense of positive change. Broadly, adaptive and active coping styles were associated with greater PTG [82].

The study found a positive relationship between deliberate rumination and PTG. This is consistent with PTG theory and results from other studies with children and adolescents [25,33,44,61]. The association could reflect that deliberate rumination involves a voluntary search to make sense of what happened and may be relevant to changing the beliefs and cognitive schemas of those who experience adverse events [61]. Deliberate rumination is therefore an important factor in facilitating PTG.

The association between PTSSs and PTG observed in youth in the present study has also been reported in previous research. Jernslett and colleagues [24] provided a detailed evaluation of this relationship, showing that PTSSs and PTG are positively associated in children and adolescents, but that PTG appears to peak at moderate levels of PTSSs, supporting a curvilinear association. Among parents and caregivers, the observed relationship was direct. Although some studies have identified a curvilinear relationship, it is possible that the COVID-19 pandemic was perceived as an uncontrollable threat [26], which may have influenced the direct association between PTSSs and PTG. Further studies are needed to better understand this relationship.

The proposed path model for PTSSs indicates that female gender is a predictor, a result consistent with some other studies [70]. Some have attributed these differences to different socialization processes or a tendency to make negative cognitive appraisals of threats [73]. That intrusive rumination, it has been found that the presence of repetitive intrusive thoughts is strongly related to PTSD [39].

Coping strategies such as cognitive avoidance, aggressive behavior, and keeping the problem to oneself, as well as unproductive, avoidant coping, in which the person suppresses and hides the worries and emotions they are experiencing, were also predictors. Such approaches tend to increase one’s challenges and associated distress, as shown by several studies evaluating the effects of engaging in aggressive behavior (including the negative influence of such behaviors on potential sources of support or connection) or of employing expressive suppression in children and adolescents (e.g., [83]). Furthermore, cognitive avoidance, that is, trying not to think about the problem, can generate more recurrent and intrusive memories of the trauma [81]. A highly stressful event for children and adolescents, such as the COVID-19 pandemic, can generate cognitions that are distressing and unwanted, such as intrusive ruminations. The person does not want to have these thoughts and tries (ineffectively) to expel them through strategies such as not dwelling on the problem; this practice causes them to spiral out of control and inhibits the individual’s ability to deploy more adaptive resources, increasing stress levels [84].

Caregivers’ posttraumatic symptoms were also predictors. This is consistent with other work supporting an association between the severity of parental trauma symptoms and childhood PTSD [85]. Studies suggest that PTSSs or PTSD may be associated with decreased levels of supervision by caregivers, who may evidence less sensitivity and responsiveness in their interactions with their children [86]. Children of caregivers with PTSD are at elevated risk for a range of internalizing and externalizing disorders and symptoms, regardless of their own exposure to potentially traumatic circumstances. Such findings have been attributed to multiple possible mechanisms or processes, including intergenerational transmission of trauma, vicarious trauma, the child’s learning through parental modeling of anxious behaviors, the impact of a distressing family environment, or diverse biological mechanisms (e.g., [85,87]).

The proposed path model for PTG suggests that coping strategies, including active solution-seeking, communication with others, and maintaining a positive attitude, are predictors. This is consistent with other research showing that adaptive coping styles are associated with greater PTG [82]. Deliberate rumination also appears relevant to PTG in the present work. This is consistent with PTG theory—as such rumination involves reflection about the event and what happened afterward, an analysis of the new situation, the search for meaning, and a reappraisal of the experience—as well as other studies with children and youth (e.g., [33,40,88].

This study acknowledges several limitations. First, its cross-sectional design prevents causal inference and limits the ability to examine trajectories of posttraumatic stress symptoms (PTSSs) and posttraumatic growth (PTG) over time. Although we refer to a “predictive model,” this denotes statistical associations rather than temporal or causal relationships. Longitudinal designs would allow for a more robust exploration of directionality and change. Second, the use of self-report instruments may introduce biases related to the subjectivity of responses. While the constructs selected are supported by prior literature, future studies should consider incorporating additional individual (child/youth or caregiver), relational, and contextual variables—such as social support—that may influence post-adversity mental health [89]. Third, several subscales—particularly those assessing rumination and coping—yielded Cronbach’s alpha coefficients below conventional thresholds. Although these instruments have been previously validated in Chilean populations and meet minimum standards for exploratory research [67], reduced reliability may have attenuated observed associations. For instance, the deliberate rumination subscale (α = 0.56) did not emerge as a significant predictor of PTSSs, possibly due to measurement error. Finally, despite efforts to recruit participants from diverse regions and school types, the sample was shaped by the availability and willingness of institutions to participate. This convenience sampling may have influenced the sociodemographic composition and limits generalizability to other educational contexts.

Future research should build on these findings through several complementary avenues. Longitudinal designs are necessary to elucidate the temporal interplay between coping strategies, rumination styles, and mental health outcomes, thereby facilitating a more precise understanding of change over time. Additionally, examining the role of social support and family functioning as potential moderators or mediators could illuminate mechanisms underlying the development of PTG and PTSD, particularly in light of the intergenerational patterns observed. Qualitative approaches may further enrich this field by exploring how children and caregivers construct meaning from adversity and how these narratives shape psychological adaptation. Finally, intervention studies focused on caregiver mental health, especially within post-crisis educational contexts, could offer promising strategies for enhancing child resilience and recovery.

This study contributes to the growing literature on post-adversity mental health by elucidating how event severity, coping responses, and rumination processes interact in shaping PTSSs and PTG among youth and their caregivers. It underscores the relevance of caregiver psychological functioning in influencing child outcomes and highlights the need for systemic approaches to mental health support. These insights are particularly valuable for professionals in clinical and educational settings, who are well-positioned to design and implement interventions that foster adaptive coping and growth in both children and adults. By identifying key psychological processes and relational dynamics, this research lays the groundwork for more targeted, context-sensitive strategies to mitigate the impact of large-scale adverse events.

## 5. Conclusions

This study is believed to be the first Chilean effort to assess PTSSs and PTG among children and adolescents and their parents or caregivers in the context of the COVID-19 pandemic. Its findings enhance understanding of the factors influencing posttraumatic stress symptoms (PTSSs) and posttraumatic growth (PTG) in children, adolescents, and their caregivers during the COVID-19 pandemic. Younger age and female gender predicted higher PTSSs in children, while caregivers’ PTSSs significantly impacted their children’s emotional well-being, highlighting intergenerational effects. Adaptive coping strategies and deliberate rumination were positively associated with PTG, whereas unproductive coping and intrusive rumination predicted greater PTSSs. No significant differences were found based on COVID-19 infection status, and family and social support emerged as important protective factors. That said, given the cross-sectional design, longitudinal studies are needed to clarify the direction of relationships among the variables. Nevertheless, these findings provide valuable insights for developing targeted interventions to support mental health and resilience in youth and their families following traumatic events and circumstances.

## Figures and Tables

**Table 1:** Descriptive statistics and internal consistency of the study variables (n = 489).

Variable	Mean	SD	α
1. Intrusive rumination	0.90	1.29	0.67
2. Deliberate rumination	3.22	1.98	0.56
3. PC—Active Solution	4.80	1.83	0.59
4. PC—Communicating to others	3.79	2.05	0.59
5. PC—Information seeking	3.83	2.12	0.68
6. PC—Positive attitude	4.69	2.10	0.74
7. UPC—Indifference	2.47	1.86	0.62
8. UPC—Aggressive behavior	2.70	2.13	0.76
9. UPC—Keeping the problem to oneself	4.85	2.52	0.85
10. AI—Cognitive avoidance	3.03	1.62	0.61
11. AI—Behavioral avoidance	4.27	2.04	0.65
12. Posttraumatic symptoms—youth	22.75	13.83	0.81
13. Posttraumatic growth—youth	17.66	6.23	0.91
14. Subjective severity of the event—caregivers	5.29	2.63	0.75
15. Posttraumatic symptoms—caregivers	8.56	6.73	0.88
16. Posttraumatic growth—caregivers	24.06	14.02	0.94

Note: PC = Problem-focused coping strategies. UPC = Unproductive coping strategies. Some variable names have been shorted for this table.

**Table 2:** Two-tailed Pearson’s r correlation between main study variables (correlations between coping strategies are omitted) (n = 489).

	9	10	11	12	13	14	15
1. PC—Active Solution	−0.05	0.27 ***	−0.08	0.41 ***	−0.01	−0.04	0.11 *
2. PC—Communicating to others	0.00	0.19 ***	−0.15 ***	0.39 ***	−0.03	−0.15 ***	−0.02
3. PC—Information seeking	0.06	0.31 ***	−0.09 *	0.40 ***	−0.05	−0.12 **	0.01
4. PC—Positive attitude	−0.08	0.18 ***	−0.26 ***	0.48 ***	−0.05	−0.08	0.00
5. UPC—Indifference	−0.08	−0.17 ***	0.09 *	−0.19 ***	−0.02	0.53 ***	−0.07
6. UPC—Aggressive behavior	0.16 ***	−0.01	0.42 ***	−0.21 ***	0.04	0.64 ***	0.04
7. UPC—Keeping the problem to oneself	0.17 ***	0.04	0.41 ***	−0.31 ***	0.08	0.71 ***	0.10 *
7. UPC—Cognitive avoidance	0.12 **	0.16 ***	0.26 ***	0.04	0.12 **	0.68 ***	0.11 *
8. UPC—Behavioral avoidance	0.18 ***	0.23 ***	0.21 ***	0.08	0.05	0.67 ***	0.06
9. Intrusive rumination	-	0.34 ***	0.51 ***	0.00	0.09 *	0.18 ***	0.09 *
10. Deliberate rumination		-	0.18 ***	0.31 ***	0.05	0.07	0.00
11. PTSSs—youth			-	−0.28 ***	0.08	0.44 ***	0.14 ***
12. PTG—youth				-	−0.07	−0.21 ***	0.02
13. SSE					-	0.08	0.37 ***
14. PTSSs—caregiver						-	0.35 ***
15. PTG—caregiver							-

PC = problem-focused coping; UPC = unproductive coping; SSE = Subjective severity of event (caregiver); PTSS = Posttraumatic stress symptomatology; PTG = Posttraumatic growth; * *p* < 0.05; ** *p* < 0.01; *** *p* < 0.001.

**Table 3:** Final model after Hierarchical Multiple Linear Regression to predict PTSSs in children (n = 489).

Model		Unstandardized Coefficients		Standardized Coefficients	t-Value
		B	SE	*β*	
Step 1	R^2^ = 0.03. ΔR^2^ = 0.03, *p* < 0.001				
	(Constant)	34.13	3.94		8.658
	Age	−0.72	0.30	−0.107	−2.380 *
	Sex	−3.72	1.15	−0.146	−3.247 ***
Step 2	R^2^ = 0.47. ΔR^2^ = 0.04, *p* < 0.001				
	(Constant)	12.68	3.67		3.453
	Age	−0.06	0.24	−0.009	−0.262
	Sex	−1.70	0.87	−0.067	−1.963 *
	Intrusive rumination	4.25	0.40	0.397	10.538 ***
	Deliberate rumination	0.26	0.27	0.037	0.971
	PC—Active solution	0.37	0.32	0.049	1.172
	PC—Communicating to others	−0.28	0.33	−0.041	−0.846
	PC—Information seeking	0.11	0.33	0.017	0.330
	PC—Positive attitude	−1.16	0.27	−0.176	−4.331 ***
	UC—Indifference	−0.12	0.28	−0.017	−0.436
	UC—Aggressive behavior	1.57	0.24	0.241	6.460 ***
	UC—Keeping the problem to oneself	1.06	0.23	0.193	4.686 ***
	UC—Cognitive avoidance	1.17	0.41	0.136	2.882 **
	UC—Behavioral avoidance	−0.27	0.33	−0.039	−0.808
Step 3	R^2^ = 0.48. ΔR^2^ = 0.01, *p* < 0.152				
	(Constant)	13.61	3.81		3.575
	Age	−0.10	0.24	−0.014	−0.392
	Sex	−1.71	0.87	−0.067	−1.978 *
	Intrusive rumination	4.21	0.40	0.394	10.438 ***
	Deliberate rumination	0.25	0.27	0.036	0.930
	PC—Active solution	0.38	0.32	0.050	1.184
	PC—Communicating to others	−0.26	0.33	−0.038	−0.780
	PC—Information seeking	0.08	0.33	0.012	0.237
	PC—Positive attitude	−1.17	0.27	−0.178	−4.361 ***
	UC—Indifference	−0.16	0.28	−0.021	−0.558
	UC—Aggressive behavior	1.52	0.24	0.234	6.230 ***
	UC—Keeping the problem to oneself	1.08	0.23	0.197	4.769 ***
	UC—Cognitive avoidance	1.15	0.41	0.134	2.821 **
	UC—Behavioral avoidance	−0.25	0.33	−0.037	−0.766
	SSE—caregivers	−0.32	0.22	−0.060	−1.434
	PTSSs—caregivers	0.16	0.09	0.080	1.905 *

PC = problem-focused coping; UC = unproductive coping; SSE = Subjective severity of event; PTSSs = Posttraumatic stress symptoms; * *p* < 0.05; ** *p* < 0.01; *** *p* < 0.001.

**Table 4:** Final model after Hierarchical Multiple Linear Regression to predict PTG in children (n = 489).

Model		Unstandardized Coefficients		Standardized Coefficients	t-Value
		B	SE	*β*	
Step 1	R^2^ = 0.01. ΔR^2^ = 0.01, *p* = 0.137				
	(Constant)	17.35	1.80		9.628
	Age	−0.02	0.14	−0.007	−0.150
	Sex	1.05	0.52	0.091	1.997 *
Step 2	R^2^ = 0.39. ΔR^2^ = 0.38, *p* < 0.001				
	(Constant)	9.42	1.79		5.266
	Age	0.05	0.12	0.015	0.394
	Sex	0.80	0.42	0.070	1.904
	Intrusive rumination	0.07	0.20	0.015	0.366
	Deliberate rumination	0.62	0.13	0.196	4.718 ***
	PC—Active solution	0.56	0.16	0.163	3.581 ***
	PC—Communicating to others	0.33	0.16	0.109	2.078 *
	PC—Information seeking	0.09	0.16	0.029	0.537
	PC—Positive attitude	0.78	0.13	0.262	5.947 ***
	UC—Indifference	0.00	0.14	0.001	0.016
	UC—Aggressive behavior	−0.25	0.12	−0.085	−2.099 *
	UC—Keeping the problem to oneself	−0.44	0.11	−0.176	−3.947 ***
	UC—Cognitive avoidance	0.14	0.20	0.035	0.685
	UC—Behavioral avoidance	−0.09	0.16	−0.030	−0.567
Step 3	R^2^ = 0.39. ΔR^2^ = 0.00, *p* = 0.250				
	(Constant)	9.69	1.86		5.207
	Age	0.05	0.12	0.015	0.399
	Sex	0.78	0.42	0.068	1.856
	Intrusive rumination	0.07	0.20	0.014	0.337
	Deliberate rumination	0.64	0.13	0.202	4.843 ***
	PC—Active solution	0.54	0.16	0.157	3.428 ***
	PC—Communicating to others	0.34	0.16	0.112	2.137 *
	PC—Information seeking	0.07	0.16	0.025	0.454
	PC—Positive attitude	0.77	0.13	0.260	5.908 ***
	UC—Indifference	0.01	0.14	0.002	0.044
	UC—Aggressive behavior	−0.25	0.12	−0.085	−2.118 *
	UC—Keeping the problem to oneself	−0.44	0.11	−0.178	−3.989 ***
	UC—Cognitive avoidance	0.15	0.20	0.038	0.731
	UC—Behavioral avoidance	−0.09	0.16	−0.030	−0.569
	SSE—caregivers	−0.14	0.09	−0.058	−1.476
	PTG—caregivers	0.02	0.02	0.049	1.250

PC = problem-focused coping; UC = unproductive coping; SSE = Subjective severity of event; PTG = Posttraumatic growth; * *p* < 0.05; *** *p* < 0.001.

**Table 5:** Standardized indirect effects, confidence intervals (CIs) and standard errors of the effect according to corrected-bias bootstrap.

Variables	Indirect Effect	CI (95%)	SE
UC—Aggressive behavior → Deliberate rumination	0.04	0.02/0.08	0.02
UC—Aggressive behavior → PTSSs	0.38	0.14/0.65	0.13
UC—Aggressive behavior → PTG	−0.15	−0.26/−0.07	0.05
UC—Cognitive avoidance → Deliberate rumination	0.03	0.00/0.07	0.02
UC—Cognitive avoidance → PTSSs	0.24	−0.01/0.63	0.16
UC—Cognitive avoidance → PTG	−0.11	−0.21/−0.04	0.04
UC—Behavioral avoidance → PTG	0.09	0.03/0.17	0.03
UC—Keeping the problem to yourself → PTG	−0.11	−0.18/−0.05	0.04
PC—Positive attitude → PTG	0.23	0.13/0.03	0.05
Intrusive rumination → PTG	−0.07	−0.30/0.14	0.11

PC = problem-focused coping; UC = unproductive coping; PTSSs = Posttraumatic stress symptoms; PTG = Posttraumatic growth.

## Data Availability

The original contributions presented in this study are included in the article. Further inquiries can be directed to the corresponding author.
